# Inhibition of Soluble Epoxide Hydrolase by Cembranoid Diterpenes from Soft Coral *Sinularia maxima*: Enzyme Kinetics, Molecular Docking, and Molecular Dynamics

**DOI:** 10.3390/md22080373

**Published:** 2024-08-17

**Authors:** Nguyen Viet Phong, Nguyen Phuong Thao, Le Ba Vinh, Bui Thi Thuy Luyen, Chau Van Minh, Seo Young Yang

**Affiliations:** 1 Department of Biology Education, Teachers College and Institute for Phylogenomics and Evolution, Kyungpook National University, Daegu 41566, Republic of Korea; ngvietphong@gmail.com; 2 Institute of Marine Biochemistry, Vietnam Academy of Science and Technology (VAST), 18 Hoang Quoc Viet, Cau Giay, Hanoi 10072, Vietnam; thaonp@imbc.vast.vn (N.P.T.); vinhrooney@gmail.com (L.B.V.); cvminh@vast.vn (C.V.M.); 3 Faculty of Pharmaceutical Chemistry and Technology, Hanoi University of Pharmacy, 13–15 Le Thanh Tong, Hoan Kiem, Hanoi 11021, Vietnam; luyenbthoaduoc@gmail.com

**Keywords:** *Sinularia maxima*, cembranoid diterpenes, soluble epoxide hydrolase, molecular docking, molecular dynamics

## Abstract

Soluble epoxide hydrolase (sEH) is essential for converting epoxy fatty acids, such as epoxyeicosatrienoic acids (EETs), into their dihydroxy forms. EETs play a crucial role in regulating blood pressure, mediating anti-inflammatory responses, and modulating pain, making sEH a key target for therapeutic interventions. Current research is increasingly focused on identifying sEH inhibitors from natural sources, particularly marine environments, which are rich in bioactive compounds due to their unique metabolic adaptations. In this study, the sEH inhibitory activities of ten cembranoid diterpenes (**1**–**10**) isolated from the soft coral *Sinularia maxima* were evaluated. Among them, compounds **3** and **9** exhibited considerable sEH inhibition, with IC_50_ values of 70.68 μM and 78.83 μM, respectively. Enzyme kinetics analysis revealed that these two active compounds inhibit sEH through a non-competitive mode. Additionally, in silico approaches, including molecular docking and molecular dynamics simulations, confirmed their stability and interactions with sEH, highlighting their potential as natural therapeutic agents for managing cardiovascular and inflammatory diseases.

## 1. Introduction

Soluble epoxide hydrolase (sEH) is a crucial enzyme that metabolizes epoxy fatty acids, such as epoxyeicosatrienoic acids (EETs), into their corresponding diols, dihydroxyeicosatrienoic acids (DHETs) [1,2]. EETs are important signaling molecules derived from arachidonic acid and play significant roles in various physiological processes, including blood pressure regulation, anti-inflammatory response, and pain modulation [3]. The role of sEH in converting EETs into less active DHETs positions it as a compelling target for therapeutic interventions [4]. sEH is predominantly expressed in the liver, kidneys, brain, and endothelium, where it influences several vital functions [5]. Given its significant role in inflammation, pain regulation, and vascular homeostasis maintenance, inhibiting sEH can elevate epoxy fatty acid levels, leading to considerable therapeutic benefits in treating conditions such as cardiovascular diseases, hypertension, and inflammatory diseases [6,7].

In recent years, there has been growing interest in identifying sEH inhibitors from natural sources, particularly marine environments [8]. Marine organisms represent a rich reservoir of bioactive compounds due to their unique metabolic pathways, which have evolved to adapt to the extreme conditions of their habitats [9]. Notably, marine-derived secondary metabolites exhibit promising sEH inhibitory activities. For instance, specific peptides and protein hydrolysates derived from marine fish have been studied for their sEH inhibitory properties, demonstrating the potential of these substances in developing nutraceuticals and pharmaceuticals targeting sEH [10,11]. Marine sponges and their associated microorganisms have also been explored for their capacity to produce sEH inhibitory compounds. These marine organisms produce secondary metabolites with diverse chemical structures, some of which exhibit significant sEH inhibitory effects [12].

Several classes of compounds have been identified as potent sEH inhibitors. Among these, terpenoids have garnered significant attention. For example, 2-oxopomolic acid, a triterpenoid isolated from *Malus domestica*, has demonstrated sEH inhibitory activity with an IC_50_ value of 11.4 ± 2.7 μM [13]. In addition, two triterpenoids with a protostane-type skeleton, 3β-hydroxy-25-anhydro-alisol F and 3β-hydroxy-alisol G, isolated from *Alisma orientale*, exhibited potential sEH inhibition with IC_50_ values of 10.06 ± 1.12 μM and 30.45 ± 2.65 μM, respectively [14]. Other protostane-type triterpenoids, such as 11-deoxy-25-anhydro alisol E and 11-deoxy alisol B, have shown sEH inhibitory effects with IC_50_ values of 5.94 ± 0.54 μM and 3.40 ± 0.57 μM, respectively [15]. These findings underscore the diversity of sEH inhibitors and emphasize the significant potential of natural products, particularly terpenoids, in this area of research.

The genus *Sinularia* comprises a diverse group of soft corals belonging to the family Alcyoniidae, within the class Octocorallia of the phylum Cnidaria [16,17]. Commonly referred to as soft corals [18], *Sinularia* species are characterized by their flexible structures and are typically found in shallow tropical marine environments [17,19,20]. The genus *Sinularia* includes over 90 described species, each exhibiting distinct morphological and chemical characteristics [20,21]. These corals serve as integral components of coral reef ecosystems, providing habitat and protection for various marine organisms. Additionally, they produce a wide range of secondary metabolites, particularly diterpenoids, which play crucial roles in chemical defense against predators and pathogens [20,21].

One notable species within this genus is *Sinularia maxima*, which has garnered attention for its distinctive chemical composition and ecological significance. *S. maxima* produces several bioactive compounds, including terpenoids such as 11β-acetoxypukalide and pukalide [22]. These compounds are not only crucial for the coral’s defense mechanisms but also have potential applications in pharmacology due to their biological activities. Studies have shown that extracts from *S. maxima* can deter feeding by reef fishes, highlighting the importance of these metabolites in the coral’s natural defense strategies [23]. Additionally, the chemical profile of *S. maxima* varies temporally, potentially influencing its susceptibility to predation and environmental stressors [23,24]. While cembranoid diterpenes from *Sinularia* species have not been previously reported as sEH inhibitors, the exploration of these compounds could reveal potential sEH inhibitory activities, enhancing our understanding of natural sEH inhibitors.

**Figure 1:**
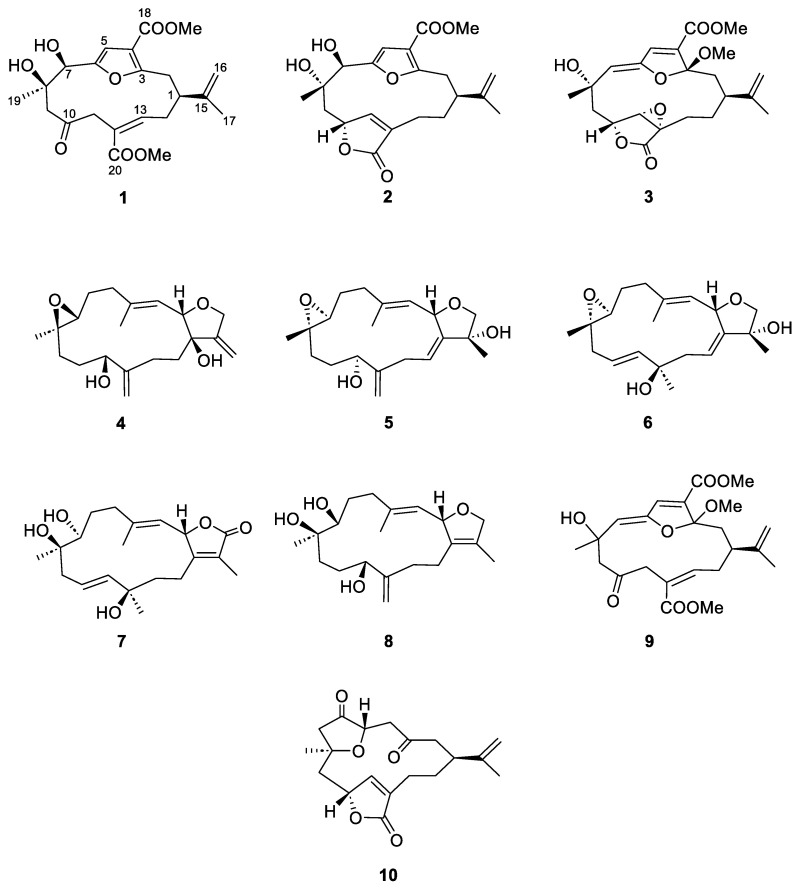
The structures of cembranoid diterpenes **1**–**10** isolated from the soft coral *Sinularia maxima*.

As part of our ongoing research to identify active compounds from the soft coral *S. maxima* [16,25], we recently reported the isolation, structural elucidation, and anti-inflammatory activities of 10 cembranoid diterpenes, including sinumaximols A–H (**1**–**8**), sethukarailin (**9**), and 5-epinorcembrene (**10**) (Figure 1). The current study offers new insights into the inhibitory activities and mechanisms of these diterpenes against sEH through a combination of in vitro assays and in silico simulations, including molecular docking and molecular dynamics (MD) studies.

## 2. Results

### 2.1. sEH Inhibitory Effects of the Isolated Cembranoid Diterpenes ***1**–**10***

The inhibitory effects of cembranoid diterpenes (**1**–**10**) on recombinant human sEH were evaluated using 3-phenyl-cyano(6-methoxy-2-naphthalenyl)methyl ester-2-oxiraneacetic acid (PHOME) as a substrate, with AUDA serving as a positive control (14.69 ± 0.07 nM) [26,27,28]. IC_50_ values indicate the concentration of each compound required to inhibit 50% of the enzyme (sEH) activity. The data revealed that most compounds, including **1**, **2**, **4**, **5**, **6**, **7**, **8**, and **10**, exhibit IC_50_ values greater than 100 μM, suggesting that they are weak inhibitors or ineffective against sEH under the tested conditions (Table 1).

In contrast, compound **3**, which contains an additional methoxy group at C-3 compared to compounds **1** and **2**, demonstrated a marked inhibitory effect against sEH, with an IC_50_ value of 70.68 ± 1.44 μM. The presence of multiple polar groups in compound **3** may enhance its binding affinity through hydrogen bonding and electrostatic interactions. Similarly, compound **9**, which also features a methoxy group at C-3, exhibited sEH inhibition with an IC_50_ value of 78.83 ± 2.26 μM. These findings suggest that the methoxy group at C-3 plays a critical role in binding to sEH.

**Figure 2:**
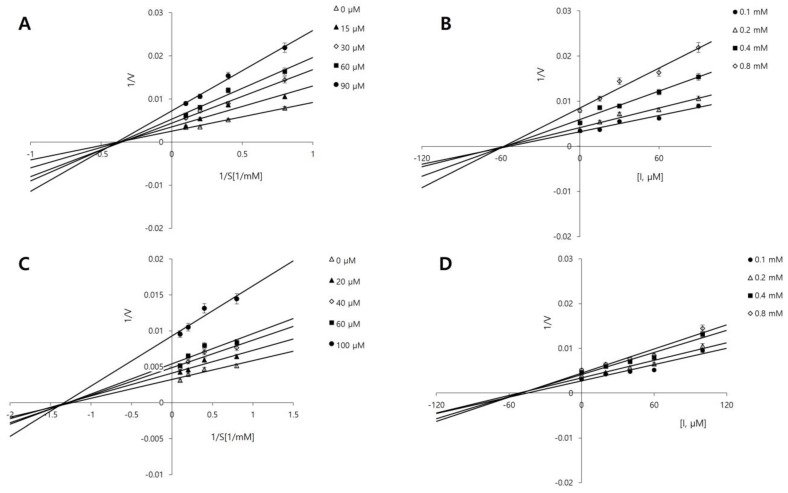
Lineweaver-Burk and Dixon plots for sEH inhibition by compounds **3** (**A**,**B**) and **9** (**C**,**D**), respectively.

The inhibition type of a compound provides insight into how it interacts with the enzyme. To further investigate the inhibition types of compounds **3** and **9**, the most potent compounds identified in this study, enzyme kinetics were analyzed using Lineweaver-Burk [29] and Dixon plots [30,31]. PHOME concentrations of 0.1, 0.2, 0.4, and 0.8 mM were used, along with compound **3** concentrations of 0, 15, 30, 60, and 90 µM, and compound **9** concentrations of 0, 20, 40, 60, and 100 µM. In the Lineweaver–Burk method, competitive and non-competitive inhibition is indicated by a series of straight lines intersecting at the same point on the y- and x-axes, respectively. As shown in Figure 2A,C, both compounds **3** and **9** exhibit non-competitive inhibition. In most cases, this implies that these inhibitors bind to an allosteric site on the enzyme rather than the active site, thereby preventing the enzyme from functioning regardless of the substrate concentration [32].

The inhibition constant (*K_i_*) is a measure of the binding affinity of the inhibitor for the enzyme, with a lower value indicating a stronger affinity. Dixon plots were used to determine the *K_i_* values for the compounds. Compounds **3** and **9** exhibited *K_i_* values of 59.44 μM and 55.03 μM, respectively (Figure 2B,D).

### 2.2. Molecular Docking

Molecular docking simulations were performed using AutoDock 4.2.6 [33] to investigate the binding interactions between sEH and compounds **3** and **9**. The structure of human sEH (entry ID: 3ANS) was obtained from the RCSB PDB database [34].

Human sEH consists of two distinct domains with separate functions: the N-terminal domain exhibits phosphatase activity, while the C-terminal domain, which encompasses both the core and cap domains, is responsible for hydrolase activity [35,36,37]. The active site of human sEH, an L-shaped hydrophobic pocket located within the protein’s core, features a narrow constriction that houses the catalytic triad (Asp335, Asp496, and His524) and stabilizing residues (Tyr383 and Tyr466) [38,39,40,41]. Additionally, the sEH enzyme possesses important allosteric sites that can be targeted for inhibition. These allosteric sites are situated outside the active site, in regions around the amino acid residues Cys423 and Cys522 [42,43]. Previous studies have demonstrated that non-competitive inhibitors can bind to these allosteric sites, disrupting enzyme function without directly competing with the substrate at the active site [42,44].

**Figure 3:**
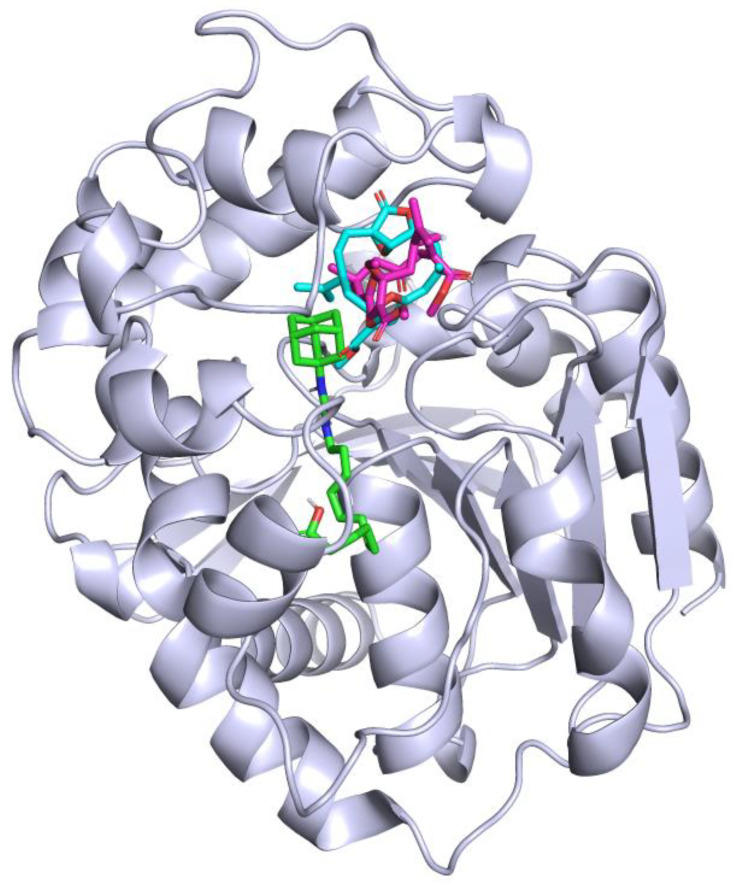
Structure of sEH illustrating the binding sites of AUDA (green), compound **3** (cyan), and compound **9** (magenta).

To optimize and validate the docking simulations, the positive control (AUDA), a known sEH inhibitor [45,46], was docked into the enzyme as a native ligand, revealing the active site. Subsequently, compounds **3** and **9** were individually docked into sEH under identical conditions. The top docking poses for each compound were clustered based on their binding energies. The primary docking site was selected from the cluster with the lowest binding energy, and alternative clusters with higher binding energies were ruled out. The binding sites of AUDA and compounds **3** and **9** on the sEH enzyme are illustrated in Figure 3.

**Figure 4:**
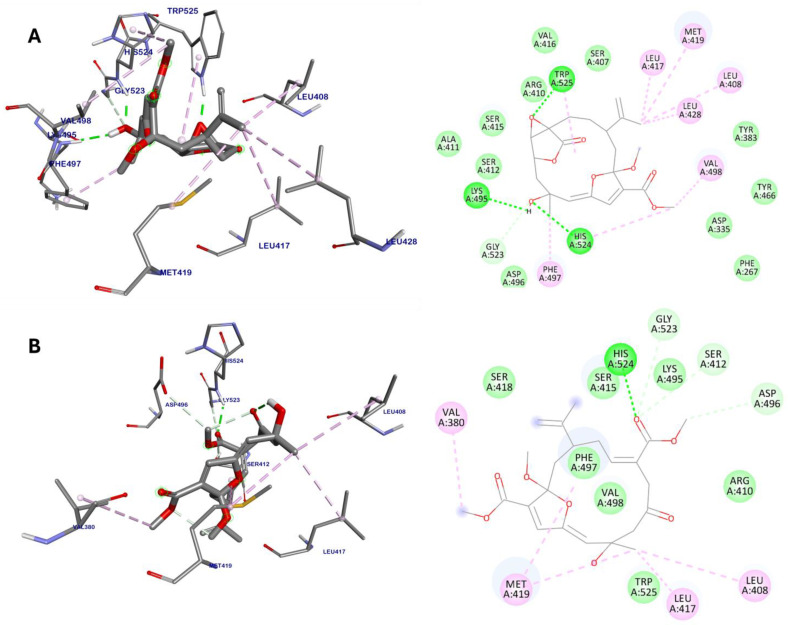
Binding interactions between sEH and compounds **3** (**A**) and **9** (**B**), along with their respective interaction maps. Green lines indicate hydrogen bonds and pink lines represent alkyl and π–alkyl interactions.

The docking results revealed that compound **3** exhibited a notably lower binding energy of −9.59 kcal/mol (Table 2), suggesting a strong interaction with sEH outside its active site, which may effectively disrupt enzyme function. Compound **3** formed hydrogen bonds with Lys495, Gly523, His524, and Trp525, and engaged in extensive van der Waals interactions with several residues, including Phe267, Asp335, Tyr383, Ser407, Arg410, Ala411, Ser412, Ser415, Val416, Tyr466, Asp496 (Figure 4A). Additionally, it established hydrophobic interactions with Leu408, Leu417, Met419, Leu428, Phe497, Val498, His524, and Trp525. These interactions strategically align with the hydrophobic and catalytic regions adjacent to the allosteric sites of sEH, which are essential for modulating enzyme activity and substrate binding.

On the other hand, compound **9** exhibited a slightly weaker binding energy of −8.94 kcal/mol compared to compound **3**. It demonstrated interactions with residues Ser412, Asp496, Gly523, and His524 through hydrogen bonds (Figure 4B). Additionally, it engaged in van der Waals interactions with residues Arg410, Ser415, Ser418, Lys495, Phe497, Val498, and Trp525. Moreover, compound **9** participated in alkyl interactions with Val380, Leu408, Leu417, and Met419. Overall, both compounds **3** and **9** exhibited similar interaction profiles, targeting key residues involved in the allosteric regulation of sEH activity. However, the differences in binding energies and specific interactions indicate that compound **3** is the more potent inhibitor, aligning with the outcomes of the in vitro studies.

Interestingly, although the methoxy group at position C-3 of the cembranoid diterpene structures was identified as an important feature for sEH inhibition in vitro, no direct interactions between the methoxy group and the sEH protein were observed for either compound **3** or compound **9**. This finding suggests that while the methoxy group may be important for enzyme inhibition, its role might be more indirect, potentially influencing the overall binding affinity or conformation of the compounds rather than directly interacting with the sEH allosteric site.

### 2.3. Molecular Dynamics

**Figure 5:**
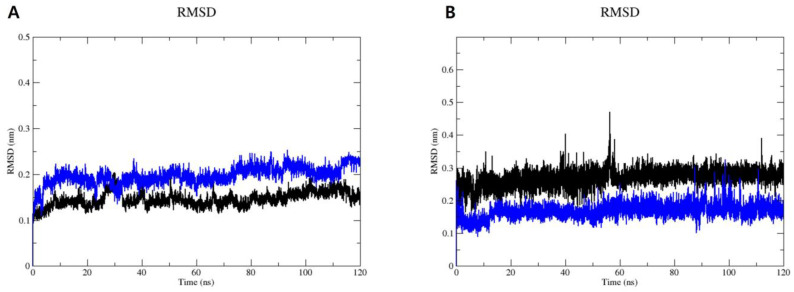
RMSD of the protein backbone (**A**) and RMSD of the ligand after least-squares fitting to the protein (**B**) of the complexes formed between sEH and compounds **3** (black) and **9** (blue).

To analyze the dynamic behavior of sEH in complexes with compounds **3** and **9**, MD simulations were conducted for 120 ns using GROMACS 2022.1 software. The root mean square deviation (RMSD) and root mean square fluctuation (RMSF) plots (Figure 5) provided further insights into the stability and flexibility of the systems. The RMSD plot of the protein backbone (Figure 5A) showed that the RMSD values for both systems fluctuated between 0.10 nm and 0.20 nm during the initial 40 ns of the MD simulations. Subsequently, both complexes maintained RMSD values of approximately 0.15 nm for the sEH–compound **3** complex and 0.20 nm for the sEH–compound **9** system until the end of the simulations. Following this, the RMSD of ligands after least-squares fitting to the protein backbone was assessed to better understand ligand binding and its impact on protein structure (Figure 5B). Throughout 120 ns of the MD simulations, compound **3** displayed an RMSD of approximately 0.23 nm, while compound **9** exhibited a slightly lower RMSD of around 0.16 nm. The low RMSD values observed for both compounds suggest that they maintain a consistent binding pose with minimal fluctuations.

**Figure 6:**
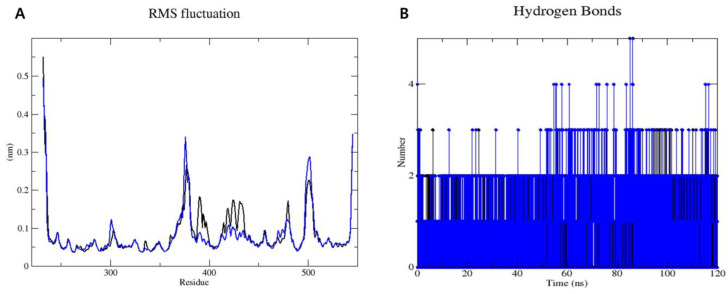
RMSF plots (**A**) and number of hydrogen bonds (**B**) for the sEH inhibition of compounds **3** (black) and **9** (blue), over the 120 ns MD trajectory.

Furthermore, the RMSF plot (Figure 6A) displayed consistent flexibility patterns among individual residues in both systems. Notably, most residues exhibited fluctuations below 0.2 nm, indicating a high level of stability throughout the simulations. However, higher fluctuations were observed at the beginning and end of the MD simulation periods. These variations can be attributed to specific residues experiencing fewer constraints within the sEH–compound **3** and sEH–compound **9** complexes, resulting in increased flexibility. Importantly, these flexible residues are located far from the binding sites of compounds **3** and **9** on sEH. Therefore, while these residues exhibit greater movement, they do not impact the binding interactions between sEH and compounds **3** and **9**.

Hydrogen bonding plays a crucial role in stabilizing protein-ligand complexes [47]. Figure 6B illustrates the hydrogen bonds formed between sEH and compounds **3** and **9** over a 120 ns MD trajectory. Both compounds **3** and **9** formed 2 to 5 hydrogen bonds with the sEH enzyme.

**Figure 7:**
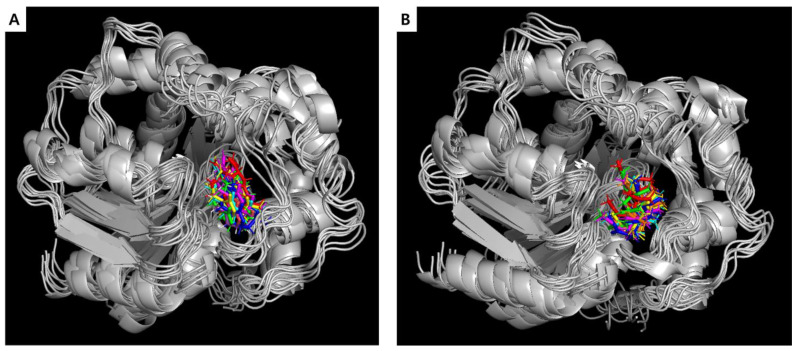
Superposition for the complexes formed between sEH and compounds **3** (**A**) and **9** (**B**) over time. Colors represent different time points: 0 ns (red), 20 ns (green), 40 ns (yellow), 60 ns (pink), 80 ns (cyan), 100 ns (orange), and 120 ns (blue).

The superposition of compounds **3** and **9** within the binding site of sEH was monitored every 20 ns; the results are presented in Figure 7. Throughout the 120 ns MD simulation, there were no significant alterations in the binding sites of these two compounds within the target enzyme sEH, indicating a stable interaction between sEH and the active compounds **3** and **9**. Comparison of these results with those from the docking studies revealed that the MD simulation consistently showed a similar binding mode throughout the 120 ns trajectory.

## 3. Discussion

Marine-derived compounds, renowned for their distinctive chemical structure and rich bioactivity, are increasingly the focus of research on enzyme inhibition [48,49,50]. Notably, soft corals, such as *S. maxima*, produce a wide array of secondary metabolites, particularly cembranoid diterpenes, which are well-known for their biological activities and potential pharmacological applications [20,51]. Our previous studies have described the isolation and structural elucidation of cembranoid diterpenes from *S. maxima*, including sinumaximols A–H (**1**–**8**), sethukarailin (**9**), and 5-epinorcembrene (**10**) [16,25]. These compounds have also been reported to possess potential anti-inflammatory activity. In this study, we evaluated the sEH inhibitory activities of these compounds. Notably, in vitro assays revealed that compounds **3** and **9** exhibited IC_50_ values of 70.68 ± 1.44 μM and 78.83 ± 2.26 μM, respectively. While these values are higher compared to some well-established sEH inhibitors [13,14,15], they still highlight the potential of compounds **3** and **9** as sEH inhibitors, especially considering their unique binding sites. Enzyme kinetics revealed that these compounds exhibit non-competitive inhibition of sEH, indicating they bind to allosteric sites distinct from the enzyme’s active site [52,53].

Virtual screening, employing molecular docking and MD simulations, is a potent strategy for the discovery and development of sEH inhibitors [54]. Molecular docking identifies potential inhibitors by assessing their binding energies and interaction patterns within binding sites [55]. In this study, molecular docking simulations were conducted to investigate the binding interactions between sEH and compounds **3** and **9**. The docking studies revealed that compounds **3** and **9** exhibited notably lower binding energies of −9.18 and −8.94 kcal/mol, respectively. Unlike well-known sEH inhibitors like AUDA, which target the active site [46], compounds **3** and **9** showed interactions with key residues at the allosteric sites of sEH. Compound **3** interacted with Lys495, Gly523, His524, and Trp525 through hydrogen bonds and engaged in van der Waals interactions with a range of residues, such as Phe267, Asp335, Tyr383, Ser407, Arg410, Ala411, Ser412, Ser415, Val416, Tyr466, and Asp496. Additionally, it formed hydrophobic interactions with Leu408, Leu417, Met419, Leu428, Phe497, Val498, His524, and Trp525. Similar interactions with key amino acid residues in the allosteric sites were also observed in the complex between compound **9** and sEH. These findings suggest that compounds **3** and **9** may inhibit sEH through a new mechanism, distinct from that of active site-binding inhibitors, potentially offering unique therapeutic benefits.

The active compounds **3** and **9**, which showed promising interactions, were further validated through MD simulations using GROMACS 2022.1 software [56] to assess their stability and conformational changes over a 120 ns period. The simulations aimed to analyze the dynamic behaviors and conformational stabilities of compounds **3** and **9** within the sEH binding site. Throughout the 120 ns simulation, both compounds exhibited minor conformational changes, as indicated by the RMSD plot. Moreover, the simulations confirmed the consistent binding modes of compounds **3** and **9** for sEH, as depicted in the RMSF plot and hydrogen bonding analysis over the simulation trajectory. Throughout the simulation period, both complexes remained robustly bound, with hydrogen bonds and van der Waals interactions contributing to the observed stability.

Investigating diterpenoids as sEH inhibitors is a relatively new approach. While other chemical classes have been extensively explored for sEH inhibition [1,27,57,58,59], marine-derived diterpenoids, particularly cembranoid diterpenes from *Sinularia maxima*, have not been studied in this context before. Our study is the first to evaluate the sEH inhibitory activity of these cembranoid diterpenes. The findings suggest that these compounds offer a new perspective on sEH inhibition, potentially expanding the collection of natural products with therapeutic applications.

In conclusion, these results underscore compounds **3** and **9** from *S. maxima* as promising sEH inhibitors, demonstrating stable interactions with the enzyme over prolonged simulations. The combination of molecular docking, MD simulations, and in vitro biochemical assays, including sEH inhibition and enzyme kinetics analysis, offers comprehensive insights into the molecular mechanisms underlying their inhibitory activity against sEH. This integrative approach provides a robust framework for the identification and characterization of marine-derived compounds with potential therapeutic applications in cardiovascular and inflammatory diseases.

However, to further substantiate these findings, additional in vivo experiments are essential to validate the inhibitory effects of compounds **3** and **9** in a physiological context. Such experimental evaluations are crucial for confirming their efficacy and safety, extremely facilitating the development and optimization of these marine-derived compounds for therapeutic use.

## 4. Materials and Methods

### 4.1. Biological Material

The soft coral *S. maxima* was collected from Nha Trang, Khanh Hoa, Vietnam, in November 2010 and authenticated by Professor Do Cong Thung (Institute of Marine Environment and Resources, VAST). The voucher specimen of *S. maxima* (accession code: SM112010_01) was deposited at the Department of Marine Medicinal Materials, Institute of Marine Biochemistry, VAST.

### 4.2. Chemicals and Reagents

sEH (human, recombinant; 10011669), PHOME (10009134), and AUDA (10007927) were purchased from Cayman Chemical (Ann Arbor, MI, USA). Bis-Tris (B9754) was purchased from Sigma-Aldrich (St. Louis, MO, USA).

### 4.3. sEH Inhibition Assay

The sEH inhibition assay was performed as described in a previous study [36], with slight modifications. In 96-well plates, separate mixtures were prepared for each test compound, containing 130 µL of sEH in Bis-Tris buffer with HCl (pH 7.0, containing 0.1% bovine serum albumin) and 20 µL of the test compound (10 mM, diluted in DMSO). Subsequently, 50 µL of PHOME, which serves as a substrate, was added to each well. The mixtures were then incubated at 37 °C, and fluorescence was monitored using the VICTOR Nivo multimode plate reader (PerkinElmer, Waltham, MA, USA) for 60 min, with excitation and emission wavelengths of 330 nm and 460 nm, respectively. AUDA, an sEH inhibitor, was used as the positive control. The inhibitory activity was calculated using the following equation:

Inhibitory activity (%) = [1 − (ΔI/ΔC)] × 100%,

where ΔI and ΔC are the fluorescence readings of the inhibitor and the control after 60 min, respectively. IC_50_ values were calculated using SigmaPlot 10.0 (Systat Software, CA, USA). Data are presented as means ± standard deviations from three independent experiments (*n* = 3, *p* < 0.05).

Enzyme kinetics were analyzed using Lineweaver–Burk and Dixon plots [29,30]. This approach helped determine the mode of inhibition and the inhibition constant (*K_i_*) at various substrate concentrations, both in the absence (0 μM) and presence of inhibitors. Data were plotted using Microsoft Excel 365.

### 4.4. Molecular Docking Simulation

Molecular docking simulations were conducted to investigate the interactions between ligand molecules and the sEH enzyme. AutoDock 4.2 (The Scripps Research Institute, La Jolla, CA, USA) [33] was employed for these simulations, following the developer’s protocol [60]. The structure of human sEH (PDB ID: 3ANS) was retrieved from the RCSB Protein Data Bank (resolution: 1.98 Å) [34]. Protein preparation, which involved adding Kollman charges (−7.0) and exporting the structure to a “.pdbqt” file, was performed using AutoDockTools 1.5.6. The 3D structures of the test compounds were constructed using Spartan’24 (Wavefunction, CA, USA).

Due to the flexibility of the macrocycles (compounds **3** and **9**), a conformational analysis was conducted to identify the most stable conformers before introducing them into the docking process. First, the conformer distribution was analyzed using Spartan’24 software with the MMFF force field and an energy cutoff of 5 kcal/mol. The obtained conformers were further optimized using the PM6 semi-empirical method, followed by the density functional theory (DFT) method at the B3LYP/6-31G(d) level in the vacuum phase (Supplementary Material). The most stable conformers were then assigned Gasteiger charges (0.0001 for compound **3** and −0.0002 for compound **9**) and exported in “.pdbqt” format for subsequent docking simulations using AutoDockTools 1.5.6.

Blind docking techniques were applied with the following parameters: grid box dimensions (x = 96, y = 96, z = 124) and grid position (x = 27.058, y = 28.677, z = 108.904). Protein-ligand interactions were investigated using a molecular docking approach with an exhaustiveness value of 24 and a maximum of 1000 binding modes. The resulting docking poses and interaction diagrams were analyzed and visualized using BIOVIA Discovery Studio Visualizer 21.1 (Dassault Systèmes, San Francisco, CA, USA) and PyMOL 2.5.4 (Schrödinger, New York, NY, USA).

### 4.5. Molecular Dynamics Simulation

MD simulations were conducted using GROMACS 2022.1 [56] with the CHARMM36 force field [61], following previously described procedures [62,63]. The system setup included periodic boundary conditions and explicit solvation in a cubic box with TIP3P water molecules. Sodium (Na^+^) and/or chloride (Cl^−^) ions were added for stability. Energy minimization was performed with a maximum force threshold of 10 kJ/mol. NVT equilibration was conducted at 300 K, followed by NPT ensemble relaxation at 1.013 bar. MD simulations were run for 120 ns, and trajectory data were analyzed using PyMOL 2.5.4 and Grace software version 5.1.22 (https://plasma-gate.weizmann.ac.il/Grace/, accessed on 10 July 2024).

## Figures and Tables

**Table 1:** sEH inhibitory effects, inhibition types, and inhibition constants (*K_i_*) of compounds **1**–**10**.

Compounds	IC_50_ (μM) ^1^	Inhibition Type ^2^	*K_i_* (μM) ^3^
**1**	>100	–	–
**2**	>100	–	–
**3**	70.68 ± 1.44	Non-competitive	59.44
**4**	>100	–	–
**5**	>100	–	–
**6**	>100	–	–
**7**	>100	–	–
**8**	>100	–	–
**9**	78.83 ± 2.26	Non-competitive	55.03
**10**	>100	–	–
AUDA ^4^	14.69 ± 0.07 nM	–	–

^1^ The values (μM) indicate 50% inhibitory effects. Data are expressed as the mean ± SD (*n* = 3). ^2^ Determined using Lineweaver-Burk plots. ^3^ Determined using Dixon plots. ^4^ Positive control.

**Table 2:** Interactions between the enzyme sEH and compounds **3**, **9**, and AUDA: binding energy, hydrogen bonds, van der Waals interactions, and hydrophobic interactions.

Compound	Binding Energy (kcal/mol)	Hydrogen Bonds	van der Waals Interactions	Hydrophobic Interactions
**3**	−9.18	Lys495 Gly523 His524 Trp525	Phe267 Asp335 Tyr383 Ser407 Arg410 Ala411 Ser412 Ser415 Val416 Tyr466 Asp496	Leu408 (alkyl) Leu417 (alkyl) Met419 (alkyl) Leu428 (alkyl) Phe497 (π−alkyl) Val498 (alkyl) His524 (π−alkyl) Trp525 (π−alkyl)
**9**	−8.94	Ser412 Asp496 Gly523 His524	Arg410 Ser415 Ser418 Lys495 Phe497 Val498 Trp525	Val380 (alkyl) Leu408 (alkyl) Leu417 (alkyl) Met419 (alkyl)
AUDA	−8.53	Asp335 Gln384 Tyr466	Phe267 Pro268 Thr360 Phe387 Leu428 Met469 Val498 Met503	Trp336 (π−alkyl) Met339 (alkyl) Phe381 (π−σ) Tyr383 (π−alkyl) Leu408 (alkyl) Met419 (alkyl) Leu499 (alkyl) His524 (π−alkyl) Trp525 (π−alkyl)

## Data Availability

Data that support the findings of this study are available in the Article.

## Supplementary Materials

The following supporting information can be downloaded at: https://www.mdpi.com/article/10.3390/md22080373/s1, Figure S1: The most stable conformers of compound **3** calculated at B3LYP/6-31G(d) level; Figure S2: The most stable conformers of compound **9** calculated at B3LYP/6-31G(d) level; Figure S3: Energy profiles, total energy, and potential energy plots for the sEH complexes with compounds **3** and **9**; Table S1: Electronic energies and Boltzmann populations for the most stable conformers of compound **3**; Table S2: Electronic energies and Boltzmann populations for the most stable conformers of compound **9**.
