# Supplementary material for: Inhibition of Soluble Epoxide Hydrolase by Cembranoid Diterpenes from Soft Coral Sinularia maxima: Enzyme Kinetics, Molecular Docking, and Molecular Dynamics
Source: Mar Drugs. 2024 Aug 17;22(8):373. doi: 10.3390/md22080373 (PMC11355918; doi:10.3390/md22080373)
Supplement: Supplementary file 1 [file marinedrugs-22-00373-s001.zip › marinedrugs-3133625-supplementary.pdf]

SUPPLEMENTARY MATERIAL

Inhibition of Soluble Epoxide Hydrolase by Cembranoid Diterpenes from Soft Coral *Sinularia maxima*: Enzyme Kinetics, Molecular Docking, and Molecular Dynamics

Nguyen Viet Phong [1,2], Nguyen Phuong Thao [2], Le Ba Vinh [2], Bui Thi Thuy Luyen [3], Chau Van Minh [2] and Seo Young Yang [1,*]

[1] Department of Biology Education, Teachers College and Institute for Phylogenomics and Evolution, Kyungpook National University, Daegu 41566, Republic of Korea; ngvietphong@gmail.com (N.V.P.), syy@knu.ac.kr (S.Y.Y.)
[2] Institute of Marine Biochemistry, Vietnam Academy of Science and Technology (VAST), 18 Hoang Quoc Viet, Cau Giay, Hanoi 10072, Viet Nam; np_thao82@yahoo.com (N.P.T.), vinhrooney@gmail.com (L.B.V.), cvminh@vast.vn (C.V.M.)
[3] Faculty of Pharmaceutical Chemistry and Technology, Hanoi University of Pharmacy, 13–15 Le Thanh Tong, Hoan Kiem, Hanoi 11021, Viet Nam; luyenbthoaduoc@gmail.com (B.T.T.L.)
* Correspondence: syy@knu.ac.kr (S.Y.Y.); Tel.: +82-53-950-5910

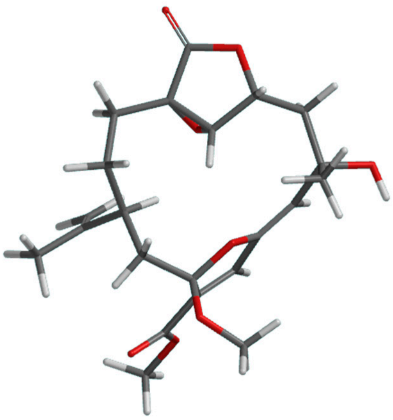

3-1

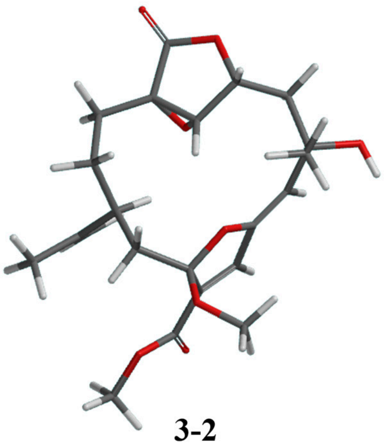

3-2

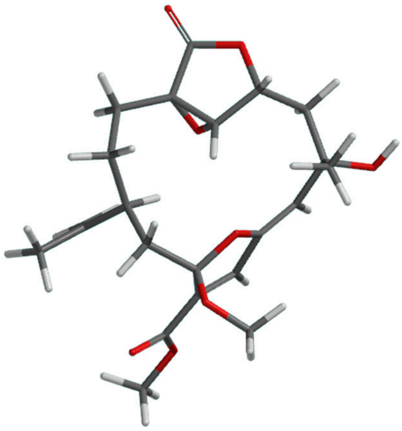

3-3

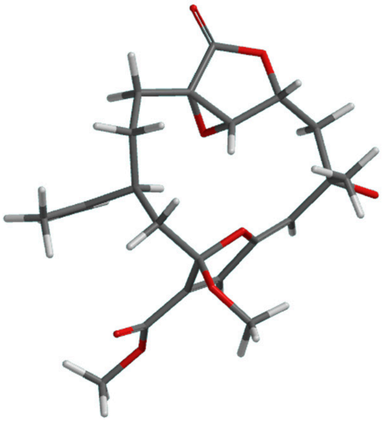

3-4

Figure S1. The most stable conformers of compound **3** calculated at B3LYP/6-31G(d) level.

Table S1. Electronic energies and Boltzmann populations for the most stable conformers of compound **3**.

Conformers	*E* (Hartree)	Boltzmann population
**3-1**	−1448.21402413	0.618335
**3-2**	−1448.21289292	0.186278
**3-3**	−1448.21255013	0.129801
**3-4**	−1448.21190644	0.065585

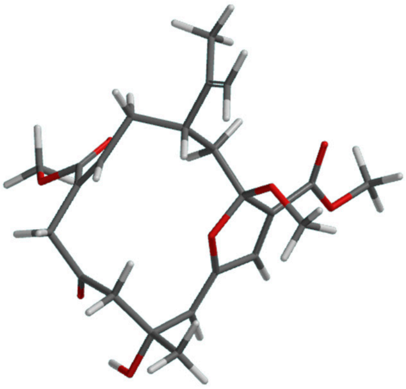

9-1

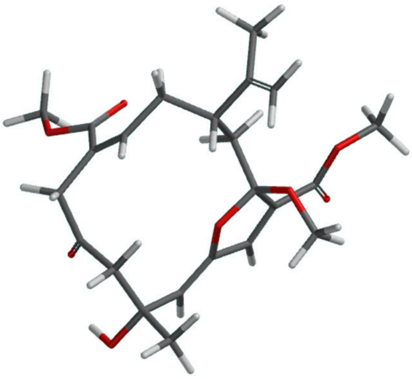

9-2

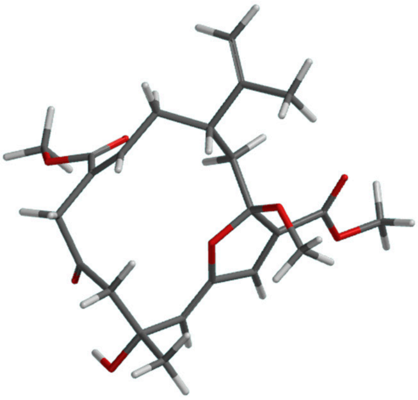

9-3

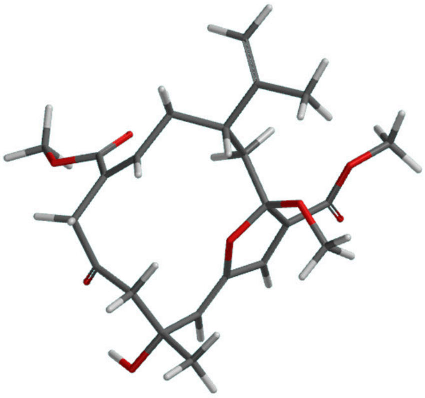

9-4

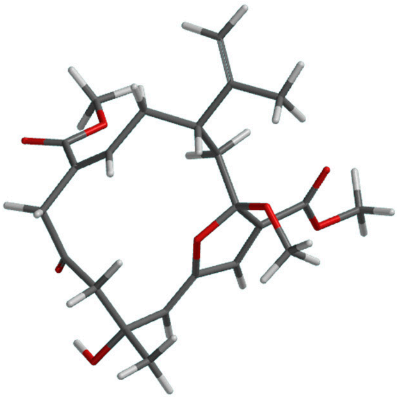

9-5

Figure S2. The most stable conformers of compound **9** calculated at B3LYP/6-31G(d) level.

Table S2. Electronic energies and Boltzmann populations for the most stable conformers of compound **9**.

Conformers	*E* (Hartree)	Boltzmann population
**9-1**	−1487.28932872	0.494028
**9-2**	−1487.28884500	0.295805
**9-3**	−1487.28811371	0.136032
**9-4**	−1487.28736338	0.061519
**9-5**	−1487.28586652	0.012615

Compound 3

Energy	Average	Err.Est.	RMSD	Tot-Drift	
Potential	-516133	38	820.964	-218.629	(kJ/mol)
**Energy**	**Average**	**Err.Est.**	**RMSD**	**Tot-Drift**	
Total Energy	-410136	38	1102.35	-214.891	(kJ/mol)

Compound 9

Energy	Average	Err.Est.	RMSD	Tot-Drift	
Potential	-516073	42	822.579	-272.639	(kJ/mol)
**Energy**	**Average**	**Err.Est.**	**RMSD**	**Tot-Drift**	
Total Energy	-410080	42	1104.2	-267.86	(kJ/mol)

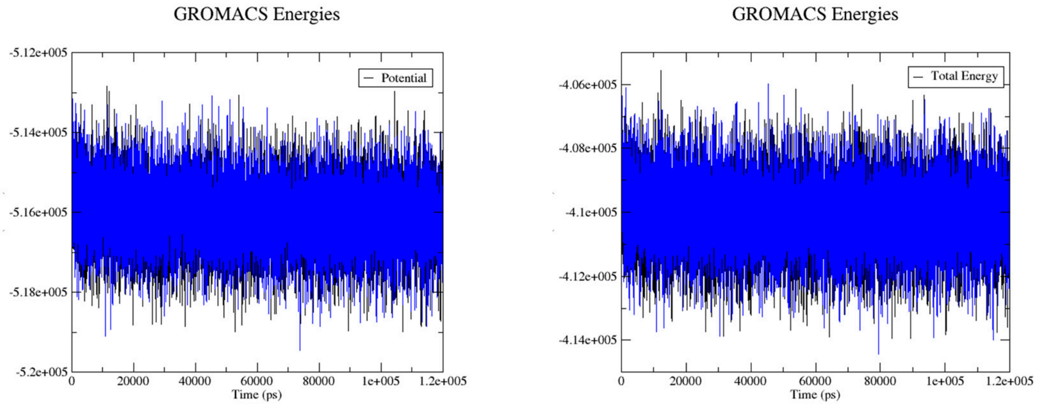

Figure S3. Energy profiles, total energy, and potential energy plots for the sEH complexes with compounds **3** (black) and **9** (blue).
